# De Novo PAX2 Mutation With Associated Papillorenal Syndrome: A Case Report and Literature Review of Penetrance and Expressivity

**DOI:** 10.7759/cureus.29026

**Published:** 2022-09-11

**Authors:** Maryam Ali, Michael Chang, Monique Leys

**Affiliations:** 1 Department of Medicine, Philadelphia College of Osteopathic Medicine - Georgia Campus, Suwanee, USA; 2 Department of Ophthalmology and Visual Sciences, West Virginia University Eye Institute, Ruby Memorial Hospital, Morgantown, USA

**Keywords:** de-novo, optic disc, no prior family history, papillorenal syndrome, nonsense mutation, pax2

## Abstract

We report the ocular findings of a Caucasian female with papillorenal syndrome (PAPRS) from a de novo *PAX2 *mutation. She presented to our clinic with early-onset end-stage renal disease. Ophthalmologic exam revealed bilateral band keratopathy, abnormal optic disc configuration, and Elschnig spots, with preserved visual acuity. Genomic sequencing revealed a heterozygous nonsense *PAX2* mutation (C > G p. (Tyr73*) at position 219 in exon 3) associated with PAPRS. Parents of the proband did not display phenotypic features of PAPRS and were confirmed to be without the *PAX2* mutation.

## Introduction

Papillorenal syndrome (PAPRS) is an autosomal dominant disorder characterized by renal and ocular abnormalities in childhood [1,2]. Fifty percent of the cases of PAPRS are related to alterations in a single copy of *PAX2* [1,3,4]. *PAX2* encodes a transcription factor critical to ophthalmic, renal, and central nervous system development [5,6]. Classic ocular findings in *PAX2*-related PAPRS relate to optic nerve irregularities, including optic nerve dysplasia and optic disc excavation [1,7]. Retinal vascular changes in the form of multiple cilioretinal vessels that exit from the disc periphery have also been reported [1,8]. Herein, we report long-term ocular findings of PAPRS due to a *PAX2* mutation in an adult without a family history of renal disease, optic disc dysplasia, or other phenotypic manifestations of PAPRS.

## Case presentation

**Figure 1:**
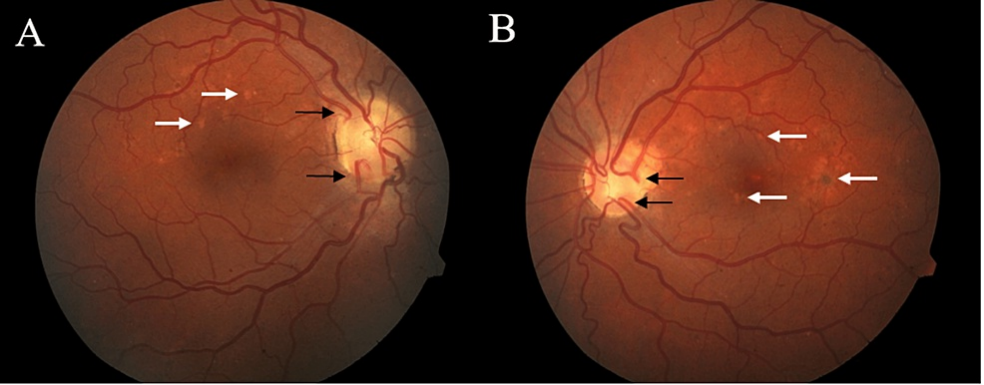
Fundoscopic exam of the right (A) and left (B) eye at initial presentation demonstrates anomalous optic nerves with multiple cilioretinal vessels (black arrows) and trace peri-papillary atrophy as well as bilateral hypopigmented lesions, suggestive of Elschnig spots (white arrows).

**Figure 2:**
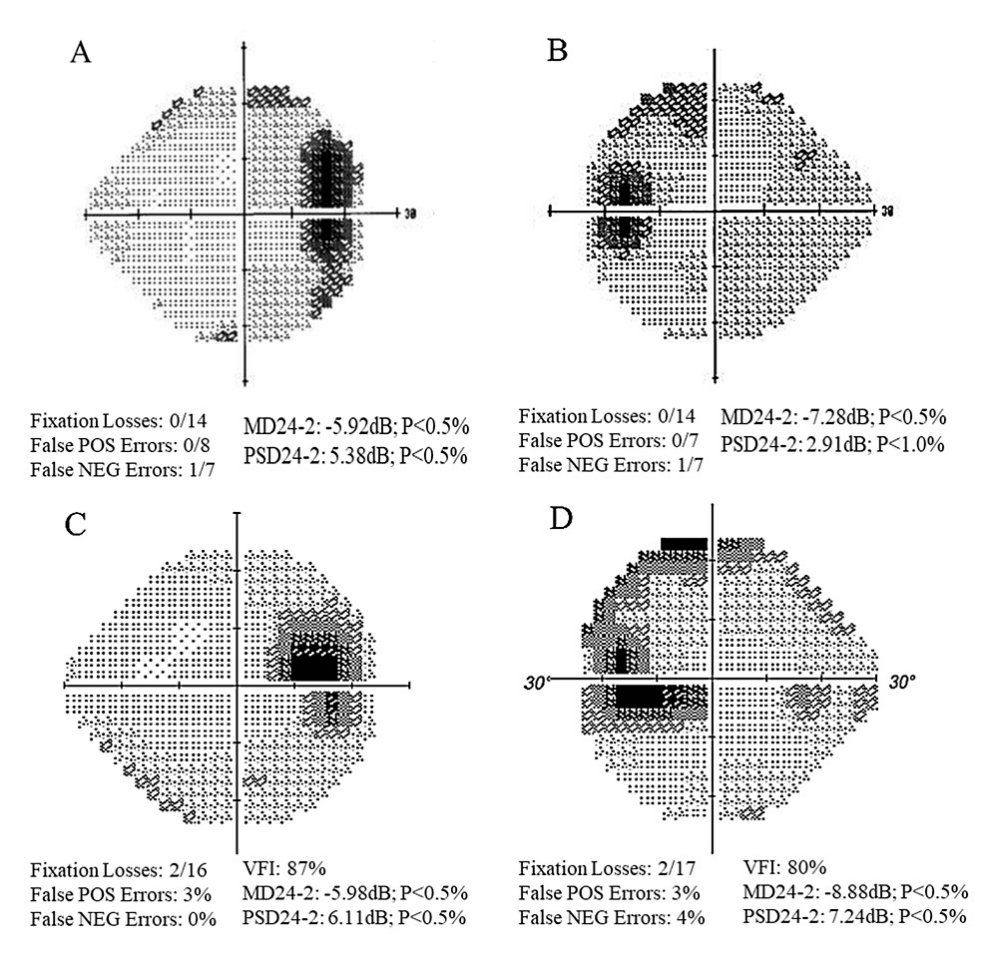
Humphrey visual fields (HVF, threshold 24-2) of right (A) and left (B) eyes at first presentation showed enlarged blind spots in both eyes with reliable indices. After 18 years of follow-up, repeat visual fields of the right (C) and left (D) eyes showed persistent enlarged blind spots with fairly reliable indices.

A 36-year-old Caucasian female presented for evaluation of maculopathy. Her past medical history was significant for dialysis-dependent end-stage renal disease (ESRD) and hypertension. She also had a known history of hypertensive retinopathy. On presentation, her visual acuity (VA) was 20/20 in both eyes. Intraocular pressure was 16 mmHg in both eyes. Anterior segment examination was unremarkable. Dilated eye exam revealed macular retinal pigment epithelial changes, Siegrist lines, and Elschnig spots in both eyes (Figure 1). Humphrey visual fields (HVF) (Humphrey Visual Field Analyzer II-I, Carl Zeiss Meditec, Inc. Dublin, CA) showed good reliability and revealed enlarged blind spots bilaterally (Figure 2A, 2B).

**Figure 3:**
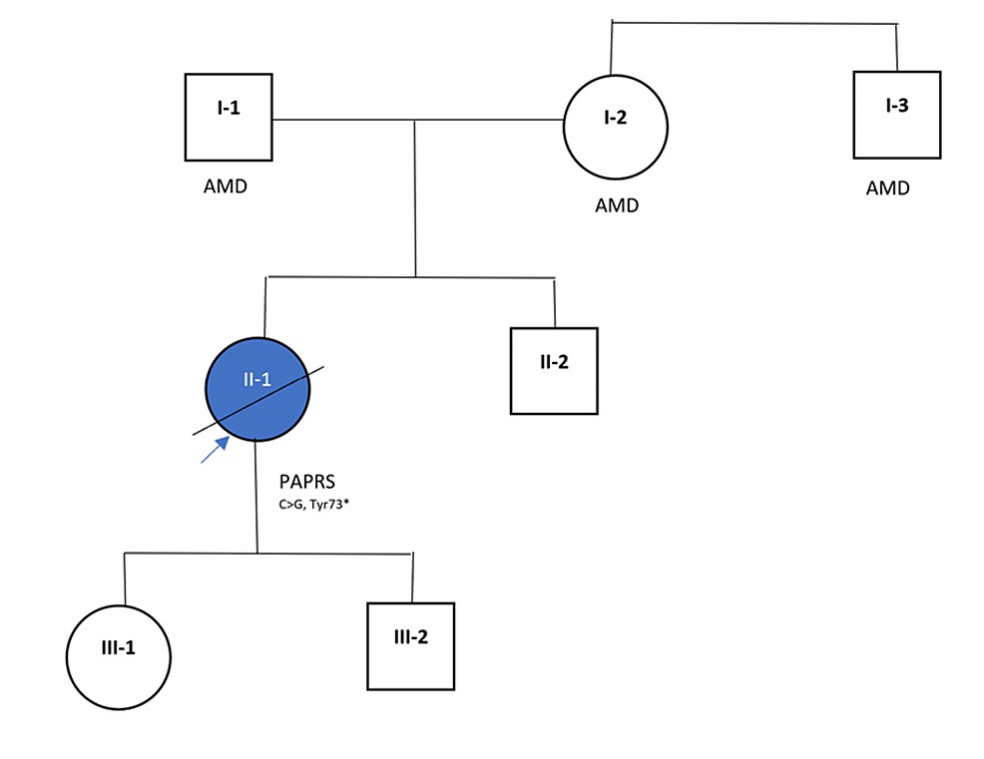
Family pedigree The proband of PAPRS is indicated with a blue arrow. PAPRS = papillorenal syndrome; AMD = age-related macular degeneration.

Family history was unremarkable for renal disease. Normal optic nerve structure on dilated eye exams was noted for the patient’s parents, uncle, and daughter. Both parents had age-related macular degeneration and had undergone a laser for retinal breaks. Her mother and maternal uncle received anti-vascular endothelial growth factor therapy for choroidal neovascularization. Her son and four grandchildren were healthy. Genomic sequencing confirmed a heterozygous nonsense *PAX2* mutation (C > G p. (Tyr73*) at position 219 in exon 3) (Blueprint Genetics, Seattle, WA, USA). Genetic testing by Invitae 330 Next-Generation Sequencing Inherited Retinal Disease panel (Spark Therapeutic Initiative, San Francisco, CA, USA) revealed that both parents did not have the *PAX2* mutation (C > G p. (Tyr73*) at position 219 in exon 3) (Figure 3).

**Figure 4:**
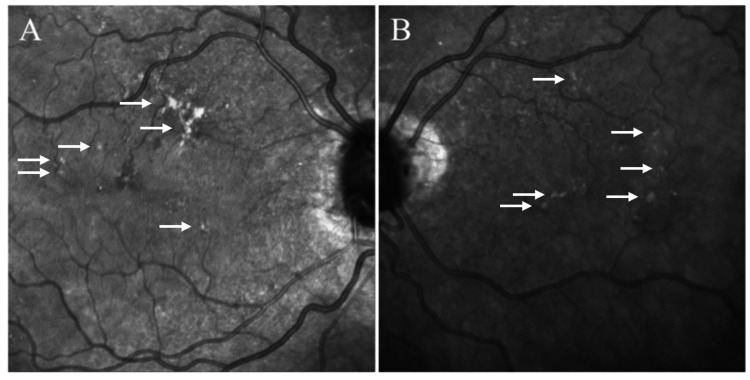
Infrared fundus photos of the right (A) and left (B) eyes demonstrate retinal pigment epithelial changes in the right eye and subretinal drusenoid deposits bilaterally (white arrow).

**Figure 5:**
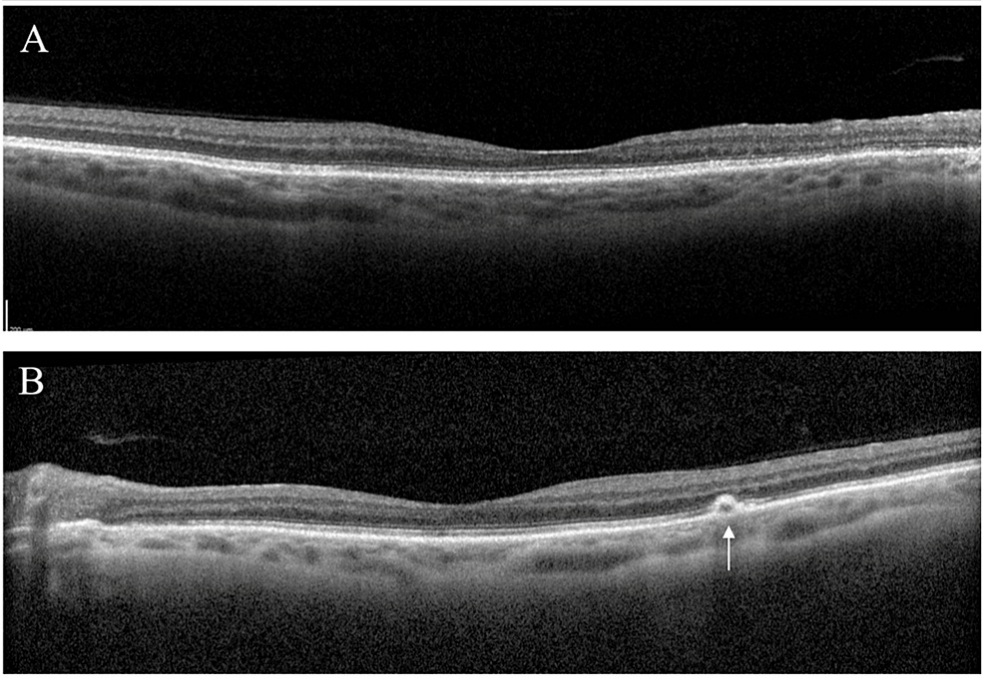
Spectral domain optical coherence tomography at final presentation shows generalized retinal thinning in the right eye (A) and a small retinal pigment epithelial detachment (white arrow) in the left eye (B).

After 18 years of follow-up, best-corrected visual acuity (BCVA) was found to be 20/20 in the right eye (OD) and 20/60 in the left eye (OS). Applanation tonometry was 22 mmHg in the right eye and 28 mmHg in the left eye. Pachymetry was 613 and 577 microns for the right and left eye, respectively. Humphrey Visual Field Analyzer II-I showed enlarging blind spots with a possible nasal step in the left eye (Figure 2C, 2D). Infrared imaging showed reticular drusen (Figure 4). Optical coherence tomography (Heidelberg Engineering, Heidelberg, Germany) revealed subretinal drusenoid deposits, generalized bilateral retinal thinning, and a small retinal pigment epithelial detachment in the left eye (Figure 5).

Systemically, our patient developed multiple complications related to renal failure, including hyperparathyroidism and dialysis-related amyloidosis. She expired at age 55 from sepsis.

## Discussion

PAPRS is a rare disorder with less than 200 cases described in the current literature [7]. Of the cases described, 50% report a heterozygous mutation in *PAX2* [1,7]. *PAX2*, located on chromosome 10, is most commonly associated with PAPRS [9]. Whereas *PAX2*-related disorders, including PAPRS, are characterized by autosomal dominant inheritance [10], 65% of pathogenic probands reporting *PAX2*-related disorders have no family history of the renal or ocular disease [11]. Additionally, several cases of PAPRS have reported no associated *PAX2* mutations, suggesting the involvement of other genes in its pathogenesis (e.g., SIX4, KIF26B, SALL4, CHD7) [4,7]. *PAX2* penetrance is very high at 99% [9], but *PAX2*-related PAPRS demonstrates significant phenotypic heterogeneity [12].

Ophthalmic manifestations of *PAX2*-related PAPRS are especially diverse. Most common ocular findings include an enlarged optic disc pit with abnormal vasculature and cilioretinal arteries, leading to mild visual impairment and superonasal visual field defects [8]. Other findings include optic nerve dysplasia, with abnormal vasculature, without functional impairment [13]. Approximately 25% of patients with PAPRS have optic nerve “colobomas,” leading to significant visual impairment [13]. VA in PAPRS can range from normal to clinical blindness. Ocular findings in *PAX2*-related PAPRS are related to the role of *PAX2* in encoding a transcription factor critical to the formation and closure of the optic fissure [14]. Penetrance of ocular abnormalities in individuals with pathogenic *PAX2* mutations is reported to be, at minimum, 77% [7]. Penetrance of renal abnormalities in pathogenic *PAX2* variants is higher at 92% [7]. Renal disease is typically the first presenting problem in patients with PAPRS [15]. Renal hypodysplasia, characterized by fewer nephrons, tissue disorganization, and smaller kidneys, is the most common renal anomaly reported in *PAX2*-related PAPRS [10]. Studies have also documented renal hypoplasia, vesicoureteral reflux, oligomeganephronia, and focal segmental glomerulosclerosis in association with PAPRS [4,7,11]. Renal pathologies can manifest in proteinuria, hypertension, and loss of renal function, resulting in ESRD [16].

To the best of our knowledge, 95 unique pathogenic *PAX2* variants have been reported [17]. Of these, over half of *PAX2* mutations are de novo and located on exons 1-5 [17]. In our patient, the *PAX2* variant resulted in a nonsense mutation, leading to a truncated protein lacking a functional domain, or loss of protein function via nonsense-mediated RNA decay. The mutation occurred in exon 3, following the substitution of cytosine for guanine, resulting in a stop codon in lieu of tyrosine. This specific *PAX2* mutation has previously been reported in two different families and classified as pathogenic. In the first family, the patient with this mutation presented with ESRD, renal hypodysplasia, and optic disc coloboma [7]. Her mother with this *PAX2* variant also had optic disc coloboma and a history of a renal transplant at 35 [7]. The patient’s maternal grandmother with this *PAX2* variant reported a renal transplant at 50 years old, but complete ophthalmic/renal findings were not available [7]. In the second family, a father and his ten-year-old daughter reported PAPRS resulting from this *PAX2* variant [18]. The daughter presented with bilateral vesicoureteral reflux, acute renal failure, left duplex kidney, left hip developmental dysplasia, optic disc anomalies in the left eye, and neuroepithelial layer detachment in the right eye. Her father had ESRD and chorioretinal atrophy in the right eye [18].

Unlike previously reported cases of this *PAX2* variant, herein, we report a case of a female with PAPRS from this mutation arising de novo. Our patient had ophthalmic manifestations related to hypertensive retinopathy from ESRD in addition to peripapillary vascular anomalies that have been documented in PAPRS. In contrast to prior cases, our patient lacked the presence of optic disc coloboma. However, visual field testing revealed enlarged blind spots that remained relatively stable after 18 years of follow-up. Although classically presenting with an autosomal dominant inheritance pattern, *PAX2* mutations can also develop de novo. In addition, due to variable penetrance and expressivity of PAPRS, family history for phenotypic abnormalities may be unrevealing.

## Conclusions

*PAX2* is within the current, widely available inherited retinal disease gene sequencing panels. Due to variable phenotypic presentation, genetic testing can be considered in patients with early-onset ESRD and ocular abnormalities to aid in early diagnosis of PAPRS and genetic counseling, in the absence of a family history of the disease.

## References

[1] Schimmenti LA (2011). Renal coloboma syndrome. Eur J Hum Genet.

[2] Devriendt K, Matthijs G, Van Damme B (1998). Missense mutation and hexanucleotide duplication in the PAX2 gene in two unrelated families with renal-coloboma syndrome (MIM 120330). Hum Genet.

[3] Rachwani Anil R, Rocha-de-Lossada C, Ayala CH, Contreras ME (2019). A new mutation in the PAX2 gene in a papillorenal syndrome patient. Am J Ophthalmol Case Rep.

[4] Okumura T, Furuichi K, Higashide T (2015). Association of PAX2 and other gene mutations with the clinical manifestations of renal coloboma syndrome. PLoS One.

[5] Torres M, Gómez-Pardo E, Gruss P (1996). Pax2 contributes to inner ear patterning and optic nerve trajectory. Development.

[6] Nornes HO, Dressler GR, Knapik EW, Deutsch U, Gruss P (1990). Spatially and temporally restricted expression of Pax2 during murine neurogenesis. Development.

[7] Bower M, Salomon R, Allanson J (2012). Update of PAX2 mutations in renal coloboma syndrome and establishment of a locus-specific database. Hum Mutat.

[8] Parsa CF, Silva ED, Sundin OH (2001). Redefining papillorenal syndrome: an underdiagnosed cause of ocular and renal morbidity. Ophthalmology.

[9] Bower M, Eccles M, Heidet L, Schimmenti LA (2011). Clinical utility gene card for: renal coloboma (papillorenal) syndrome. Eur J Hum Genet.

[10] Schimmenti LA, Pierpont ME, Carpenter BL, Kashtan CE, Johnson MR, Dobyns WB (1995). Autosomal dominant optic nerve colobomas, vesicoureteral reflux, and renal anomalies. Am J Med Genet.

[11] Bower MA, Schimmenti LA, Eccles MR (2007). PAX2-related disorder. GeneReviews® [Internet].

[12] Deng H, Zhang Y, Xiao H (2019). Diverse phenotypes in children with PAX2-related disorder. Mol Genet Genomic Med.

[13] Dureau P, Attie-Bitach T, Salomon R, Bettembourg O, Amiel J, Uteza Y, Dufier J-L (2001). Renal coloboma syndrome. Ophthalmology.

[14] Otteson DC, Shelden E, Jones JM, Kameoka J, Hitchcock PF (1998). Pax2 expression and retinal morphogenesis in the normal and Krd mouse. Dev Biol.

[15] Ng B, De Silva S, Bindra M (2021). Papillorenal syndrome: a systemic diagnosis not to be missed on funduscopy. BMJ Case Rep.

[16] Xiong HY, Shi YQ, Zhong C (2022). Detection of de novo PAX2 variants and phenotypes in Chinese population: a single-center study. Front Genet.

[17] (2022). The Human Variome Project: PAX2 (paired box 2). https://databases.lovd.nl/shared/individuals/PAX2.

[18] Wang X, Shao J, Liao P (2019). Report of papillorenal syndrome in a family and literature review. Chin J Nephrol.

